# Profiling miRNAs in nasopharyngeal carcinoma FFPE tissue by microarray and Next Generation Sequencing

**DOI:** 10.1016/j.gdata.2014.08.005

**Published:** 2014-08-27

**Authors:** Jin Peng, Yanjun Feng, Gabriel Rinaldi, Paul Levine, Samantha Easley, Elizabeth Martinez, Salman Hashmi, Nader Sadeghi, Paul J. Brindley, Jason P. Mulvenna, Jeffrey M. Bethony, Jordan L. Plieskatt

**Affiliations:** a Department of Microbiology, Immunology and Tropical Medicine, School of Medicine and Health Science, George Washington University, Washington DC, USA; b Research Center for Neglected Diseases of Poverty, School of Medicine and Health Science, George Washington University, Washington DC, USA; c Department of Epidemiology and Biostatistics, The George Washington University School of Public Health and Health Services, Washington DC 20037, USA; d Department of Pathology, School of Medicine and Health Science, George Washington University, Washington DC, USA; e Medical Faculty Associates, The George Washington University, Washington DC 20037, USA; f School of Biomedical Sciences, Faculty of Medicine and Biomedical Sciences, University of Queensland, Brisbane, Australia; g Infectious Diseases Program, QIMR Berghofer Medical Research Institute, Brisbane, Queensland, Australia

**Keywords:** miRNA, Biomarker, Microarray, Nasopharyngeal carcinoma, RNA-Seq

## Abstract

Nasopharyngeal carcinoma (NPC) is a non-lymphomatous, squamous-cell carcinoma that occurs in the epithelial lining of the nasopharynx. Nasopharyngeal carcinoma has a geographically well-defined distribution worldwide, with the highest prevalence in China, Southeast Asia, and Northern Africa. Symptoms of nascent NPC may be unapparent or trivial, with diagnosis based on the histopathology of biopsied tissue following endoscopy of the nasopharynx. The tumor node metastasis (TNM) staging system is the benchmark for the prognosis of NPC and guides treatment strategy. However, there is a consensus that the TNM system is not sufficiently specific for the prognosis of NPC, as it does not reflect the biological heterogeneity of this tumor, making another biomarker for the detection of NPC a priority. We have previously reported on different approaches for microRNA (miRNA) biomarker discovery for Formalin Fixed Paraffin Embedded (FFPE) NPC tissue samples by both a targeted (microarray) and an untargeted (small RNA-Seq) discovery platform. Both miRNA discovery platforms produced similar results, narrowing the miRNA signature to 1–5% of the known mature human miRNAs, with untargeted (small RNA-Seq approach) having the advantage of indicating “unknown” miRNAs associated with NPC. Both miRNA profiles strongly associated with NPC, providing two potential discovery platforms for biomarker signatures for NPC. Herein, we provide a detailed description of the methods that we used to interrogate FFPE samples to discover biomarkers for NPC.

Specifications

**Table:** 

Organism/cell line/tissue	*Homo sapiens*
Sex	5 males 3 females
Sequencer or array type	Agilent human miRNA microarray Illumina Genome Analyzer IIx
Data format	Raw and processed
Experimental factors	Tumor vs. adjacent tissue in FFPE
Experimental features	Biomarker analysis in NPC FFPE tissue between tumor and control nasopharynx.
Consent	IRB approved
Sample source location	Washington DC, United States

## Direct link to deposited data

Microarray deposited data can be found here: https://www.ncbi.nlm.nih.gov/geo/query/acc.cgi?acc=GSE46172

RNA-Seq deposited data can be found here: http://www.ncbi.nlm.nih.gov/sra/?term=SRP029599

## Experimental design, materials and methods

### Experimental cases

Case and control tissue including sample characteristics are presented in Table 1 (and in detail in [1]). In brief, four formalin fixed paraffin-embedded (FFPE) tissues from cases of histologically confirmed non-keratinizing NPC and four FFPE cases of normal nasopharyngeal tissue were obtained from the biological repository in the Department of Pathology of The George Washington University Hospital, Washington, DC. Tissue sections from FFPE were reviewed by two independent pathologists (E.M. and S.E.) to confirm the diagnosis as shown in [1]. FFPE preparation, hematoxylin and eosin (H&E) staining, and representative images have also been previously reported [1]. It should also be noted that the SRA project submission contains four additional samples (Accession: SRX345915, SRX345913, SRX345913 and SRX345909). These samples reference a survey of serum pools from NPC positive and control individuals discussed in [1] but not further referenced herein.

### RNA isolation

Total RNA was isolated from 2 × 10 μm sections from each FFPE case using the miRNeasy FFPE kit (Qiagen) [1]. RNA concentration, purity, and integrity (RIN) were determined by spectrophotometry (Nanodrop 1000) and the Agilent 2100 Bioanalyzer using the Agilent RNA 6000 Nano and small RNA kits. Purified RNA was stored at <− 50 °C.

Yields of total RNA derived from FFPE were approximately 100 ng/μm with 260/280 and 260/230 ratios of ~ 2.0 and ~ 1.9, respectively. Analysis on the Agilent Bioanalyzer indicated that the samples were enriched for small RNA species with integrity (RNA Integrity Number or RIN) values of two to three. Though typically indicative of RNA degradation, the robustness of miRNAs in these FFPE tissue [2] and reports from other groups [3] that RIN values have negligible effect on miRNA results enabled us to consider this purified RNA suitable for further analysis by microarray and RNA sequencing.

### Microarray, data normalization and analysis

All eight samples underwent analysis via microarray (Table 1). Total RNA isolated from each FFPE case was labeled and hybridized to an Agilent human miRNA microarray (miRBase Release 16.0) and scanned [1]. The intensities of each sample were transferred to digital data and log_2_ transformed using Agilent Feature Extraction (V.10.7). Raw data files in text (.txt) format were analyzed with Agilent GeneSpring software (GX 12.6) [4]. A total of 1205 human and 144 human viral microRNAs were used from miRBase v16.0.

To analyze the differentially expressed miRNAs, quantile normalization was performed to standardize these data across the samples. Raw data (thresholded and log base 2 transformed) were filtered by expression values (20.0–336133.0) with at least two out of the eight samples having values within the cut-off range to remove very low signal values and background influence. The four tumor samples were grouped and analyzed against the four control samples by unpaired Student's *t*-test with a *p*-value cut-off of 0.05 (*p*-value obtained by Asymptotic analysis) and a fold-change cut-off of 2.0. Hierarchical clustering was then performed [1] using the Euclidean distance metric and Centroid linkage rule. We identified 35 significantly dysregulated miRNAs, including four Epstein–Barr Virus (EBV) miRNAs and 31 human miRNAs (13 down-regulated and 18 up-regulated) [1]. These analyses were conducted again for this manuscript to verify their reproducibility. In addition, the miRNA signatures were compared to the recently released miRBase (v 19.0) with its up-date the miRNA nomenclature (Table 2) than in the original publication of these data, which used miRBase (v 16.0) [1].

**Fig. 1:**
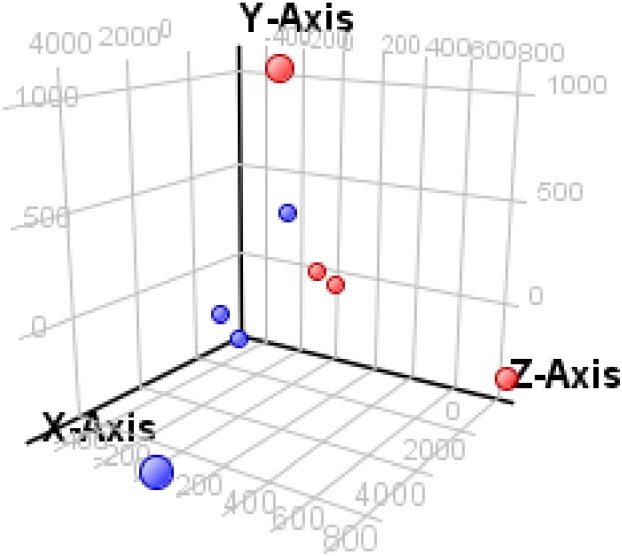
3D principal components analysis (PCA). PCA analysis of FFPE samples analyzed via microarray. Control FFPE tissue is denoted by red circles and NPC FFPE tissue is denoted by blue. No significant clustering was observed.

Significance analysis was completed using GeneSpring [4] as detailed below:

1) A new *project* was created, followed by a new *experiment*, and *miRNA* was selected for analysis type, followed by the *data import wizard* for workflow type.
2) In *New miRNA Experiment Steps*, the raw intensity files were uploaded. The selected technology was set to 31181_v16_0 and no baseline transformation was performed. The threshold raw signals were set to 1.0 and *quantile* was chosen as the normalization algorithm along.
3) In the *Experiment Setup*, the samples were grouped into four tumor and four control cases under the *Experiment Grouping* option. While further interpretations may be created depending on analysis requirements, in this case experimental parameters “tumor/control” (categorical) were set up.
  The condition tumor and control were selected *and Non*-*Averaged for the Average Over Replicates* in Conditions. *Detected* and *Not Detected* were selected and *Compromised in Use Measurements* Flagged.
4) Quality control: The correlation coefficient value of all samples was > 0.7 and therefore all the samples were used in further analysis. Further, 3D Principle Components Analysis (PCA) scores and plotting were used to determine any association among the samples (Fig. 1). It was noted that paired samples did not exhibit more significant clustering than non-paired (NPC/Control tissue) in the analysis (Fig. 1 and Hierarchical clustering [1]). In *Filter by Expression*, the right entity and interpretation were selected and filtered by raw data value. The lower cut-off value of the interest range was set to 20 and at least two out of eight samples had values within this range.
5) In *Analysis*, the condition was set as tumor versus control, tested by *t*-test unpaired, and an asymptotic *p*-value was computed without correction. The fold change cut-off was > 2.0 and analyzed under pairs of conditions with tumor compared to control. Hierarchical clustering analysis of differentially expressed genes from all samples was conducted on both entities and the conditions by normalized intensity values using Euclidean distance metric and Centroid linkage rule.

### Small RNA sequencing

Small RNA sequencing was performed on five of the same samples used in microarray analysis (Table 1) of the three samples used in the previous analysis (control samples 341E and 11311E and tumor sample 341B) omitted due to the exhaustion of total RNA purified from the small tissue areas available for the study. Total RNA derived from the FFPE was subjected to Ribo-Zero Pretreatment using Ribo-Zero rRNA Removal Kit (Epicentre) as described by the manufacturer and in [1]. Library preparation and sequencing have been described in further details in [1]. Briefly, Illumina libraries were constructed from 1 μg of total RNA using the TruSeq Small RNA Sample Kit (Illumina). Libraries were subjected to quality control prior to sequencing using an Agilent 2100 BioAnalyzer and concentration determination using PicoGreen (Invitrogen). The Illumina Genome Analyzer IIx was used to perform the sequencing by Expression Analysis, A Quintiles Company (Durham, NC).

### Sequencing processing: alignment, mapping and annotation

Initial processing was performed using both FastqMcf and FastQC both of which can be accessed at http://code.google.com/p/ea-utils/wiki/FastqMcf and http://www.bioinformatics.babraham.ac.uk/projects/fastqc.

After adaptor removal and quality filtering, ~ 28 million reads were aligned to the human (UCSC hg19) and Human herpes virus 4 (Epstein–Barr virus or EBV) genome (NCBI NC_007605.1) and miRNA counts generated for each sample [1]. Both miRDeep 2.0.0.5 [5] and miRExpress 2.0 [6] were used to generate counts, and each provided comparable results, with over 50% of the reads mapping to miRNAs in either the human or EBV genomes (Table 3). Identification of known miRNAs was based on miRBase Release 19 [7], with an alignment identity of 1%, a tolerance range of 4, and a similarity threshold of 0.8 [1]. In total, using miRDeep and miRExpress, 984 and 847 human and EBV miRNAs were identified, respectively, with a count per million greater than one in at least two of the samples.

**Fig. 2:**
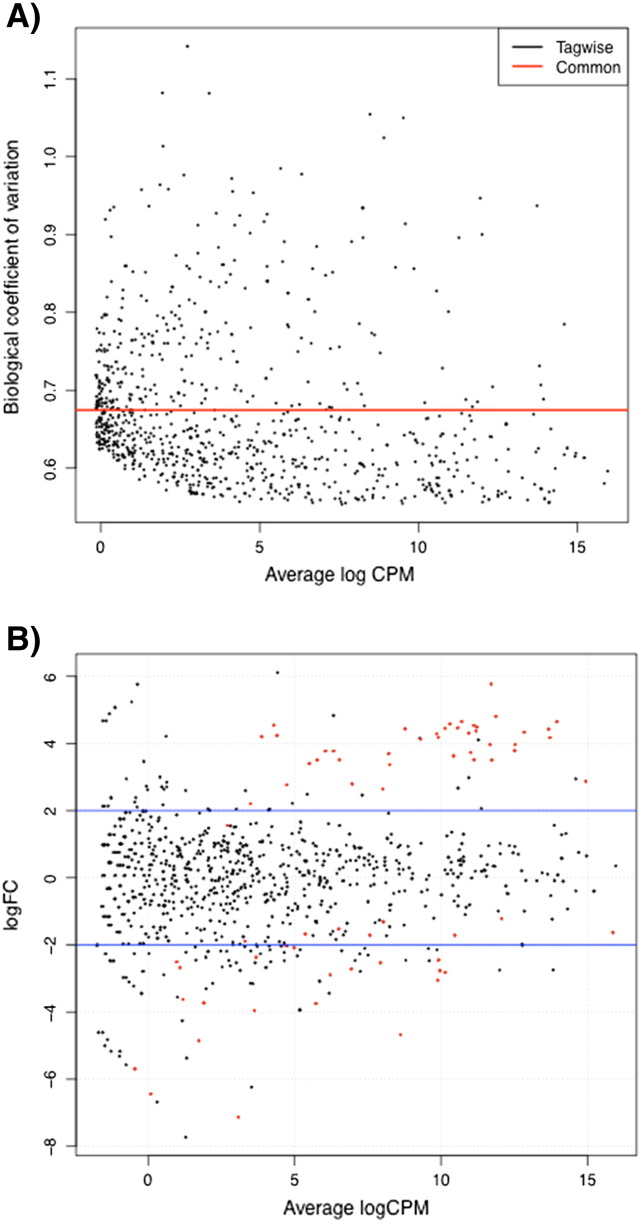
RNA-sequencing output of five NPC FFPE samples. (A) Biological coefficient of variation reported against average log CPM. Red common trend line indicates the BCV of 67%. (B) logFC reported versus average log CPM. Log fold change of two is indicated by bracketed blue lines. Red dots indicate human miRNAs identified as significant (*p* value < 0.05).

Using EdgeR [8], a binomial distribution was used to compare the independent analyses from miRDeep and miRExpress [1]. The biological coefficient of variation (BCV) was used to estimate the variability across the dataset and plotted via the *plotBCV* function (Fig. 2A), with a common dispersion of 67% indicating a relatively high dispersion of gene expression levels. Given that this was an observational study on independent NPC cases using NPC tumors of different histological grades, such a value would not be considered atypical. Using the function *plotsmear* in EdgeR, log-fold changes were plotted against log-cpm (Fig. 2B). Using EdgeR, 99 dysregulated miRNAs were identified in NPC tumor tissue versus control tissue samples.

### Comparison of datasets

**Fig. 3:**
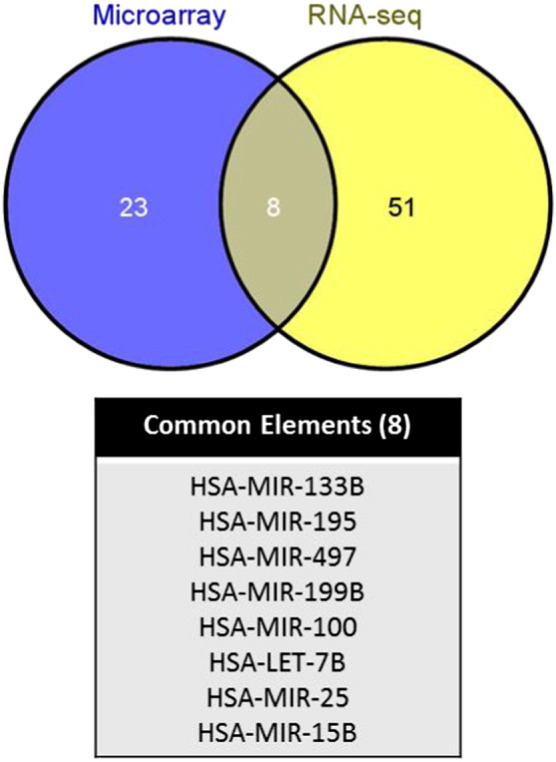
Top common human miRNAs illustrated [11] as detected in corresponding independent analyses from both microarray and RNA-Seq. A total of eight common miRNAs were highlighted across both methods under the statistical cut-offs previously described [1].

Both targeted (microarray) and untargeted (small RNA-Seq) approaches were extensively compared in our previously published manuscript [1]. While only eight dysregulated human miRNAs were identified in both the microarray and RNA-Seq analysis (Fig. 3) as statistically significant, the overall datasets were comparable. All but three miRNAs identified by microarray as significantly dysregulated were also identified by RNA-Seq, albeit not as significant [1]. In addition, these miRNAs also showed a similar dysregulation: i.e. if identified as up-regulated by microarray, they were also identified as up-regulated by small RNA-Seq [1].

## Figures and Tables

**Table 1:**
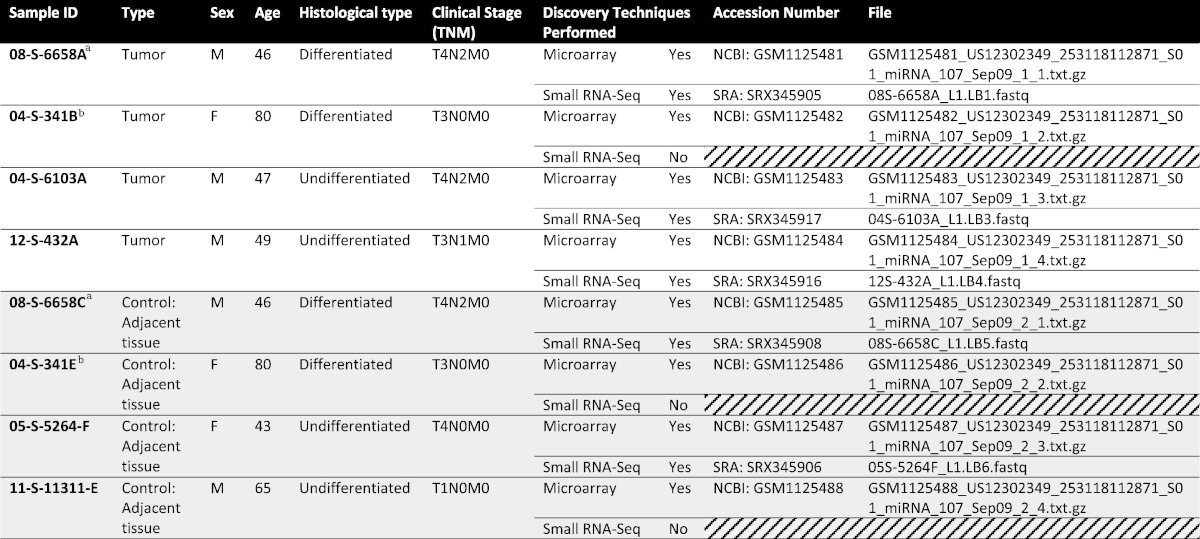
List of the raw data files deposited to NCBI GEO and SRA with accession numbers. Further details on the FFPE sample set in [1] with histological type, TNM staging [9] and WHO classification [10].

^a,b^Denotes those from the same patient (i.e. paired NPC/Control tissue samples).

**Table 2:** Microarray miRNA expression analysis between tumor and control NPC FFPE tissue using unpaired Student's *t*-test (*p*-value < 0.05 and fold-change > 2.0). In this repeated analysis by GeneSpring updated nomenclature found in miRBase v19.0 was utilized to update the sample set found in [1]. Thirty-five miRNAs were dysregulated comprising four EBV specific miRNAs.

Systematic name	FC	Log FC	Regulation	Active sequence	Chromosome	miRBase accession no.
ebv-miR-BART4-3p	99.86	6.64	Up	ACACCTGGTGCCTAC	–	MIMAT0009204
ebv-miR-BART5-5p	69.01	6.11	Up	CGATGGGCAGCTATA	–	MIMAT0003413
ebv-miR-BART6-3p	92.81	6.54	Up	TCTAAGGCTAGTCCGAT	–	MIMAT0003415
ebv-miR-BART6-5p	99.86	6.64	Up	CCTATGGATTGGACCAA	–	MIMAT0003414
hsa-let-7b-5p	− 2.09	− 1.06	Down	AACCACACAACCTACTACC	chr22	MIMAT0000063
hsa-miR-100-5p	− 2.92	− 1.55	Down	CACAAGTTCGGATCTACGG	chr11	MIMAT0000098
hsa-miR-106b-5p	2.13	1.09	Up	ATCTGCACTGTCAGCAC	chr7	MIMAT0000680
hsa-miR-125b-5p	− 2.20	− 1.14	Down	TCACAAGTTAGGGTCTC	chr11	MIMAT0000423
hsa-miR-1260a	2.27	1.18	Up	TGGTGGCAGAGGTGG	chr14	MIMAT0005911
hsa-miR-1274a_v16.0	2.94	1.56	Up	TGGCGCCTGAACAG	chr5	MIMAT0005927
hsa-miR-1274b_v16.0	2.44	1.29	Up	TGGCGCCCGAACA	chr19	MIMAT0005938
hsa-miR-1275	− 3.38	− 1.76	Down	GACAGCCTCTCCCC	chr6	MIMAT0005929
hsa-miR-130b-3p	2.18	1.12	Up	ATGCCCTTTCATCATTGC	chr22	MIMAT0000691
hsa-miR-133b	− 688.84	− 9.43	Down	TAGCTGGTTGAAGGGGACC	chr6	MIMAT0000770
hsa-miR-141-3p	4.93	2.30	Up	CCATCTTTACCAGACAG	chr12	MIMAT0000432
hsa-miR-149-5p	7.11	2.83	Up	GGGAGTGAAGACACGGAG	chr2	MIMAT0000450
hsa-miR-15b-5p	2.05	1.03	Up	TGTAAACCATGATGTGCTGC	chr3	MIMAT0000417
hsa-miR-17-3p	8.90	3.15	Up	CTACAAGTGCCTTCAC	chr13	MIMAT0000071
hsa-miR-17-5p	2.44	1.29	Up	CTACCTGCACTGTAAGC	chr13	MIMAT0000070
hsa-miR-18a-5p	13.99	3.81	Up	CTATCTGCACTAGATGCA	chr13	MIMAT0000072
hsa-miR-195-5p	− 6.00	− 2.59	Down	GCCAATATTTCTGTGCTGC	chr17	MIMAT0000461
hsa-miR-196b-5p	47.26	5.56	Up	CCCAACAACAGGAAACTACC	chr7	MIMAT0001080
hsa-miR-199a-3p	− 2.65	− 1.41	Down	TAACCAATGTGCAGACTACT	chr1	MIMAT0000232
hsa-miR-199b-5p	− 3.96	− 1.99	Down	GAACAGATAGTCTAAACACTGG	chr9	MIMAT0000263
hsa-miR-203a	41.64	5.38	Up	CTAGTGGTCCTAAACATT	chr14	MIMAT0000264
hsa-miR-20a-5p	2.18	1.13	Up	CTACCTGCACTATAAGCAC	chr13	MIMAT0000075
hsa-miR-221-3p	4.19	2.07	Up	GAAACCCAGCAGACAATGT	chrX	MIMAT0000278
hsa-miR-25-3p	2.28	1.19	Up	TCAGACCGAGACAAGTGC	chr7	MIMAT0000081
hsa-miR-3138	− 2.31	− 1.21	Down	ACTCCCTCTACCTCACT	chr4	MIMAT0015006
hsa-miR-3651	3.06	1.62	Up	TCATGTACCAGCGACC	chr9	MIMAT0018071
hsa-miR-3663-3p	− 2.55	− 1.35	Down	GCGCCCGGCCT	chr10	MIMAT0018085
hsa-miR-451a	− 6.12	− 2.61	Down	AACTCAGTAATGGTAACGGTTT	chr17	MIMAT0001631
hsa-miR-486-5p	− 3.90	− 1.96	Down	CTCGGGGCAGCTCA	chr8	MIMAT0002177
hsa-miR-497-5p	− 5.17	− 2.37	Down	ACAAACCACAGTGTGCTG	chr17	MIMAT0002820
hsa-miR-93-5p	3.28	1.71	Up	CTACCTGCACGAACAG	chr7	MIMAT0000093

**Table 3:** Total reads derived from small RNA-Seq of five FFPE samples. Total numbers of reads mapped to miRNA are reported.

Sample ID	Type	Total reads	miRNA mapped	Unmapped reads	%mapped
08-S-6658A^a^	Tumor	5,609,415.00	2,569,276.00	3,040,139.00	46%
04-S-6103A	Tumor	4,338,399.00	2,258,744.00	2,079,655.00	52%
12-S-432A	Tumor	5,354,631.00	3,236,216.00	2,118,415.00	60%
08-S-6658C^a^	Control	6,265,846.00	3,755,819.00	2,510,027.00	60%
05-S-5264-F	Control	6,533,346.00	4,262,886.00	2,270,460.00	65%
	*Total*	*28*,*101*,*637.00*	*16*,*082*,*941.00*	*12*,*018*,*696.00*	*57*%

a Denotes from the same patient (i.e. paired NPC/Control tissue samples).
